# Serotype diversity and antimicrobial susceptibility profiles of *Actinobacillus pleuropneumoniae* isolated in Italian pig farms from 2015 to 2022

**DOI:** 10.1186/s13567-024-01305-x

**Published:** 2024-04-09

**Authors:** Flavia Guarneri, Claudia Romeo, Federico Scali, Simona Zoppi, Nicoletta Formenti, Antonio Marco Maisano, Salvatore Catania, Marcelo Gottschalk, G. Loris Alborali

**Affiliations:** 1 Istituto Zooprofilattico Sperimentale della Lombardia e dell’Emilia Romagna, Brescia, Italy; 2 Globe Institute, University of Copenhagen, Copenhagen, Denmark; 3 Istituto Zooprofilattico Sperimentale del Piemonte, Liguria e Valle D’Aosta, Turin, Italy; 4 Istituto Zooprofilattico Sperimentale delle Venezie, Verona, Italy; 5 Faculty of Veterinary Medicine, University of Montreal, Saint-Hyacinthe, QC Canada

**Keywords:** Swine disease, porcine pleuropneumonia, antimicrobial resistance, APP serotyping

## Abstract

*Actinobacillus pleuropneumoniae* (APP) is a bacterium frequently associated with porcine pleuropneumonia. The acute form of the disease is highly contagious and often fatal, resulting in significant economic losses for pig farmers. Serotype diversity and antimicrobial resistance (AMR) of APP strains circulating in north Italian farms from 2015 to 2022 were evaluated retrospectively to investigate APP epidemiology in the area. A total of 572 strains isolated from outbreaks occurring in 337 different swine farms were analysed. The majority of isolates belonged to serotypes 9/11 (39.2%) and 2 (28.1%) and serotype diversity increased during the study period, up to nine different serotypes isolated in 2022. The most common resistances were against tetracycline (53% of isolates) and ampicillin (33%), followed by enrofloxacin, florfenicol and trimethoprim/sulfamethoxazole (23% each). Multidrug resistance (MDR) was common, with a third of isolates showing resistance to more than three antimicrobial classes. Resistance to the different classes and MDR varied significantly depending on the serotype. In particular, the widespread serotype 9/11 was strongly associated with florfenicol and enrofloxacin resistance and showed the highest proportion of MDR isolates. Serotype 5, although less common, showed instead a concerning proportion of trimethoprim/sulfamethoxazole resistance. Our results highlight how the typing of circulating serotypes and the analysis of their antimicrobial susceptibility profile are crucial to effectively manage APP infection and improve antimicrobial stewardship.

**Supplementary Information:**

The online version contains supplementary material available at 10.1186/s13567-024-01305-x.

## Introduction

*Actinobacillus pleuropneumoniae* (APP) is a Gram-negative bacterium frequently associated with porcine pleuropneumonia. Particularly, APP infections may lead to necrotizing and haemorrhagic pneumonia which usually affects only one diaphragmatic lobe and is accompanied by significant pulmonary oedema [1]. The acute form of the disease is highly contagious and often fatal, resulting in significant economic losses for pig farmers due to production losses and antimicrobial treatment costs [2].

*Actinobacillus pleuropneumoniae* strains can be classified according to their biotypes or serotypes. Traditionally, APP was distinguished into two biotypes: biotype I, which is nicotinamide adenine dinucleotide (NAD) dependent, and biotype II which can synthesize NAD in the presence of specific pyridine nucleotides or their precursors [3]. Isolates belonging to biotype II seem to induce milder infections than those belonging to 1 [4]. Currently, 19 APP serotypes are recognised based on capsular antigens [5, 6]. Serotype classification tends to be more informative than biotype classification because serotypes greatly differ in pathogenicity and are characterised by different combinations of the four main APP toxins. Apx IV has haemolytic but no cytotoxic activity, and is present in all serotypes, making it suitable for diagnosis [7, 8]. Apx I–III largely determine, through their cytotoxicity and haemolytic activity, the virulence of the serotype and one or two of the three are present in all serotypes [2]. The difference in virulence among the different serotypes can be partly explained by the differential production of the Apx toxins; however, this relationship is not always straightforward [9]. For example, serotype 8 could be considered “mild” based on its toxins combination, but is actually the most virulent variant of APP in the UK [10]. Similarly, serotype 7, which produces only one cytotoxin and is traditionally considered among the least virulent strains, is the causative agent of half of the clinical outbreaks of APP in Canada [11]. The distribution of serotypes involved in outbreaks in different regions of the world is indeed radically different: strains of a specific serotype may typically be highly virulent in a region, while conversely be low in virulence in another region [1]. Although APP outbreaks are currently a relatively minor problem in North America, they remain a challenge for swine production in Italy and other European countries, as well as in Asia and Latin America [1].

Strategies to prevent porcine pleuropneumonia are mainly based on external biosecurity measures to avoid the introduction of new serotypes/strains by carrier pigs (usually gilts in breeding herds and growers in fattening herds), as well as internal biosecurity measures to interrupt infection chains, for example, by age-segregated rearing [12] and litter-segregated rearing [13]. Although several vaccines against APP are available, their efficacy is limited by the presence of numerous serotypes, mostly in the case of bacterins. Moreover, the pathogenic mechanisms and virulence factors (other than toxins) of APP are not fully known yet [14]. To control the disease after the onset of the first clinical symptoms, early treatment of diseased animals with effective antimicrobials is therefore still a necessity [15], and metaphylactic treatment of exposed pigs could be required to minimize losses, all the more so because, in acute and hyperacute disease, pigs often die without showing any typical clinical signs [2]. On the other hand, reducing the use of antimicrobials in animal production is crucial for public health. Current EU legislation on veterinary medicines requires metaphylaxis to be used only in scenarios where the risk of transmission is high, and no suitable alternatives are available [16].

Proper use of antimicrobials for the treatment of APP infections requires knowledge of the susceptibility of the infecting strain. Differences in antimicrobial resistance (AMR) patterns have been observed not only among different serotypes but also over time [4]. Several studies have also reported differences in AMR patterns depending on the country in which they were conducted. Nevertheless, different antibiotic susceptibility testing techniques have been used and the methods are not fully comparable. Only a limited number of studies have been conducted using the microdilution method (MIC) [4, 15, 17, 18]. Knowledge of APP resistance profiles is necessary not only for clinical but also for epidemiological purposes. Antimicrobial resistance (AMR) is on the rise and there is a need to closely monitor antimicrobial susceptibility, to observe trends over time and ensure the long-term effectiveness of antimicrobials [19].

This study aimed to retrospectively evaluate the serotype diversity and antimicrobial susceptibility of APP strains in northern Italy, analysing trends over 8 years (2015–2022), including multi-drug resistance (MDR).

## Materials and methods

### Isolation of bacterial strains

A total of 572 APP isolates were collected from 2015 to 2022 at the Istituto Zooprofilattico Sperimentale della Lombardia e dell’Emilia Romagna (IZSLER), which routinely receives samples from swine farms located in Northern Italy. All the strains were recovered from the lungs of pigs that died of acute respiratory diseases and were submitted to the institute for analysis. Bacterial strains were isolated on an agar plate with *Staphylococcus aureus* (ATCC 25923) added to the centre of the plate and incubated at 37 °C with 5% CO_2_.

### Serotyping

The serotypes were determined by molecular methods. Bacterial DNA was extracted via lysis boiling (98 °C, 10 min) and subsequent amplification was performed by a multiplex PCR of specific sequences of genes associated with the prevalent APP serotypes of diagnostic interest: 1, 2, 3, 4, 5, 6, 7, 8, 9/11, 12, 13, 14, 17, and 18. The analysis consisted of two multiplex PCR: the first identifies the species-specific apxIV toxin and serotypes 2, 4, 5, 7, 8, 9/11, and 13. The second identifies the serotypes 1, 3, 6, 12, 14, 17, and the nadV gene that identifies biotype II [5, 6].

The Qiagen Multiplex PCR Plus kit (Qiagen) was used according to the manufacturing instructions and all the primers used have been described previously [5]. Multiplex PCR reactions mix were composed as follows: 12.5 μL of Multiplex PCR master mix (2×), 2.5 μL of Coral Dye and 1.25 μL of primer mix 10× (0.5 μL of each primer) 2 μL of genomic DNA and 6.75 μL of RNase DNase-free water to a final volume of 25 μL. The cycling parameters were as follows: an initial denaturation at 95 °C for 5 min; 30 cycles at 95 °C for 30 s, 62 °C for 90 s, and 72 °C for 60 s; a final extension at 72 °C for 15 min. The amplified PCR products were subjected to electrophoresis at a 1.5% agarose gel in 1× TBE buffer.

### Determination of antimicrobial resistance

APP isolates were subjected to antimicrobial susceptibility testing by the microdilution method. Minimum Inhibitory Concentrations (MICs) were determined by broth microdilution using a commercial plate (Sensititre Vizion Digital MIC, Thermo Fisher Scientific USA), that includes 11 antimicrobials belonging to nine different classes (Sensititre ITISVE3 plate, Thermo Fisher Scientific, USA).

The strains were classified as susceptible or resistant based on epidemiological cut-off values (ECOFFs) recommended by the European Committee on Antimicrobial Susceptibility Testing (EUCAST). The cut-off values for the tested antimicrobials are reported in Additional file 1.

### Statistical analysis

Variation in AMR during the study period was analysed through a set of nine mixed logistic regressions, one for each of the tested antimicrobials. Only 369 out of 572 isolates (64.5%) belonging to the most common serotypes (i.e., prevalence > 5%) were included in the analysis. In each model we included the serotype and year of each APP isolate as explanatory variables, and the farm ID as a random intercept, to account for different isolates submitted by the same farm. In all models, year^2^ was initially included to account for potential curvilinear trends, and removed from final models when non-significant (*p*-value for removal set at 0.1). The same model was applied to investigate the probability of an isolate being multidrug-resistant (MDR), i.e., resistant to at least three antimicrobial classes [20]. Post-hoc comparisons between serotypes were performed through t-tests on the difference of least square means, applying Tukey correction for multiple comparisons. The alpha-level for significance was set at 0.05. All the analyses were carried out in SAS/STAT 9.4 software (Copyright © 2011, SAS Institute Inc., Cary, NC, USA).

## Results

From 2015 to 2022, a total of 572 strains of APP were isolated from swine samples that were submitted to IZSLER by 337 different North Italian farms experiencing APP outbreaks. The number of samples per year ranges from a minimum of 38 in 2015 to a maximum of 86 in 2020. The 61% of the outbreaks occurred in finishers, 38% in weaners and 1% in sows. Most of the farms (66%) submitted samples from a single outbreak during the study period, 19% from two outbreaks, 7% from three outbreaks and 8% of farms submitted samples from more than three outbreaks (Additional file 2). A single isolate per outbreak was obtained: serotype analysis was performed on all the 572 strains/outbreaks, while MICs were determined for 465 of them.

**Figure 1:**
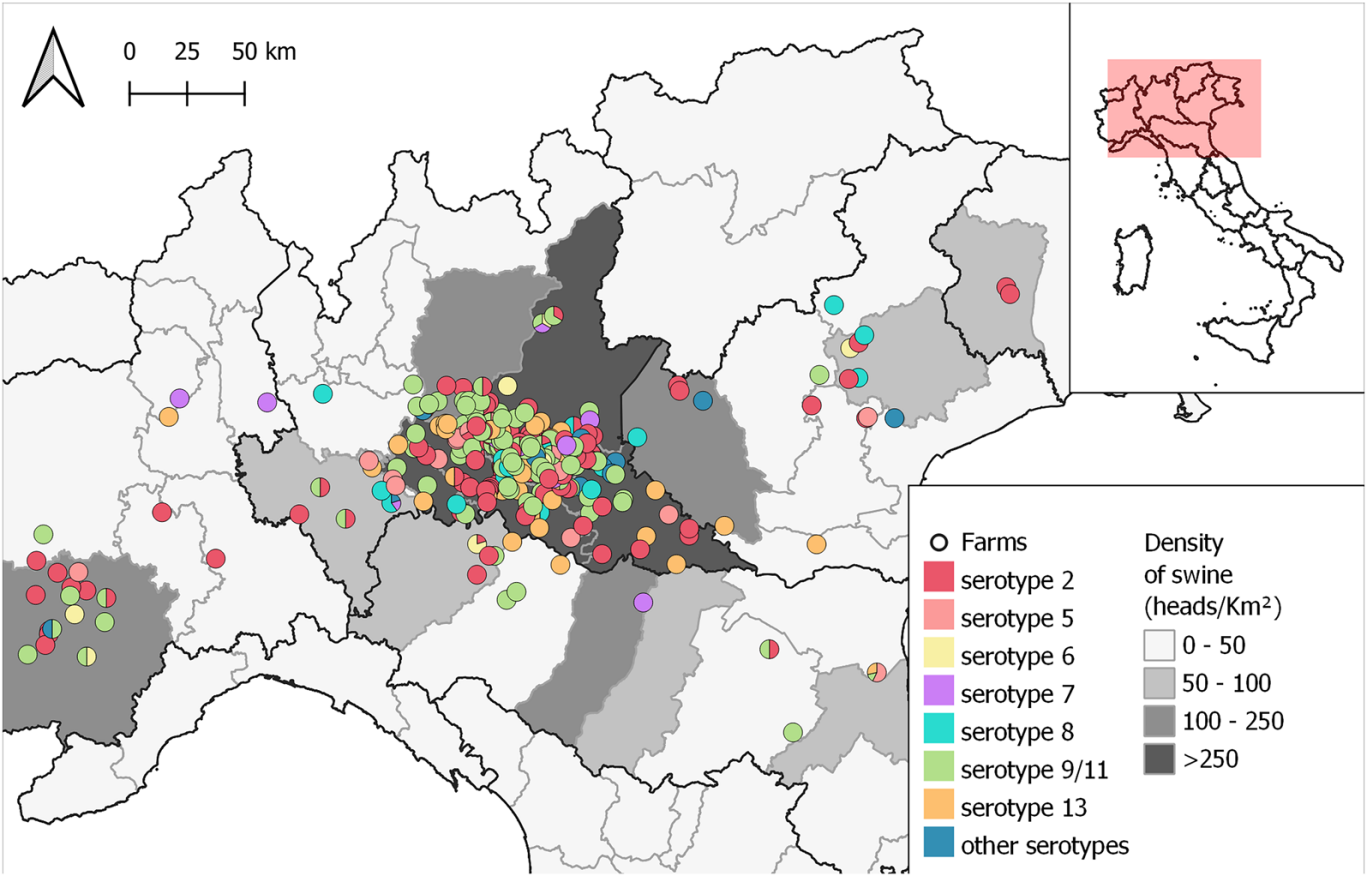
**Location of sampled pig farms.** Location of pig farms experiencing APP outbreaks during 2015–2022 and included in the present study. Circle colours and background shading represent, respectively, the serotypes isolated within each farm and the density of swine in each province, as detailed in the figure legend.

Overall, 502 (87.8%) isolates were typed successfully, while 70 were untypable. Out of the serotyped isolates, most belonged to serotypes 9/11 (39.2%) and 2 (28.1%). Serotypes 13, 5, 8, 6 and 7 were less common and other serotypes were rarely found (i.e., <1%, see Table 1 for detailed prevalence). Overall, the vast majority of the isolates (89.2%) belonged to biotype I, which was predominant in all serotypes except for serotype 13, where 70.2% of the isolates belonged to biotype II, and serotypes 2 and 3, where the few isolates all belonged to biotype II (Table 1). The geographical location of the sampled farms and the distribution of serotypes are shown in Figure 1.

**Table 1:** **Prevalence of APP serotypes**

Serotype	N	Prevalence	95% CI of the prevalence	% Biotype II
9/11	197	39.24	34.96–43.53	5.29
2	141	28.09	24.14–32.03	1.42
13	49	9.76	7.16–12.37	70.83
5	44	8.76	6.28–11.25	0
8	31	6.18	4.06–8.29	3.23
6	12	2.39	1.05–3.73	0
7	11	2.19	0.91–3.48	9.09
3	4	0.8	0.02–1.58	25.0
4	3	0.6	0–1.27	0
14	3	0.6	0–1.27	100
10	2	0.4	0–0.95	0
15	2	0.4	0–0.95	100
1	1	0.2	0–0.59	0
12	1	0.2	0–0.59	0
18	1	0.2	0–0.59	0

Prevalence and its 95% Confidence Interval of the successfully serotyped APP strains (*n* = 502) isolated from disease outbreaks occurred in north Italian swine farms from 2015 to 2022. For each serotype, the percentage of isolates belonging to biotype II is also reported.

**Figure 2:**
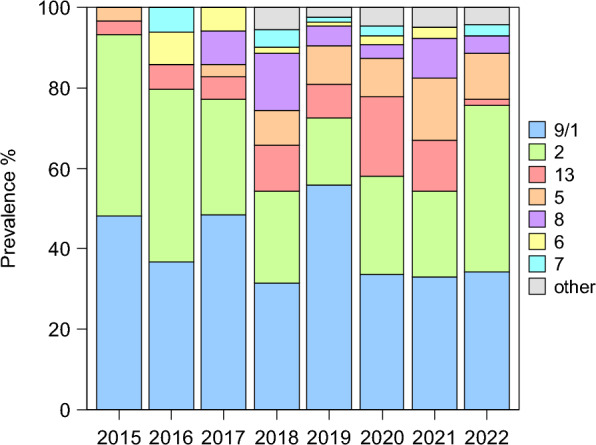
**Prevalence of APP serotypes by year.** Prevalence of serotypes by year in the APP isolates (*n* = 502) serotyped during the study period.

The prevalence of the most common serotypes varied from year to year. In particular, the prevalence of serotype 5 increased significantly over the study period (*p* = 0.012; R^2^ = 0.74), from 3.5% in 2015 to more than 10% of samples in 2020 and 2021. Serotype 8 emerged starting from 2017 (Figure 2). In general, there was an increase in the serotype diversity of circulating APP, from four different serotypes detected in 2015 to nine serotypes in 2022.

**Figure 3:**
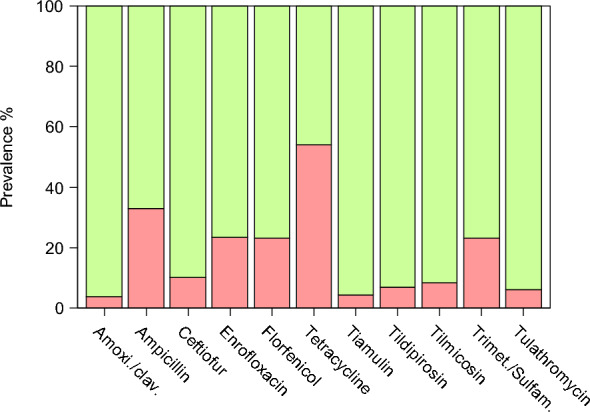
**Prevalence of AMR in APP isolates.** Resistance of the examined APP isolates (*n* = 465) to the tested antimicrobials.

Regarding AMR, 317 out of 465 (68%) of the examined isolates were resistant to at least one antimicrobial class. The most common resistances were against tetracycline (53% of isolates) and ampicillin (33%), followed by enrofloxacin, florfenicol and trimethoprim/sulfamethoxazole (23% each). Resistance to ceftiofur and tilmicosin occurred in 10% and 8% of isolates respectively. Resistance to tildipirosin (7%), tulathromycin (6%), tiamulin (5%) and amoxicillin/clavulanic (4%) was less frequent (Figure 3).

An MDR was found in 148 out of 465 (32%) of the isolates, two of which were resistant to all the nine antimicrobial classes tested. The probability of observing MDR depended on the serotype (χ^2^_4_ = 28.2; *p* < 0.001), the smallest proportion of MDR isolates was found in strains belonging to serotype 2 (17%), the biggest in serotype 9/11 (46%). No temporal trend in MDR prevalence across years was detected (*p* > 0.05).

**Figure 4:**
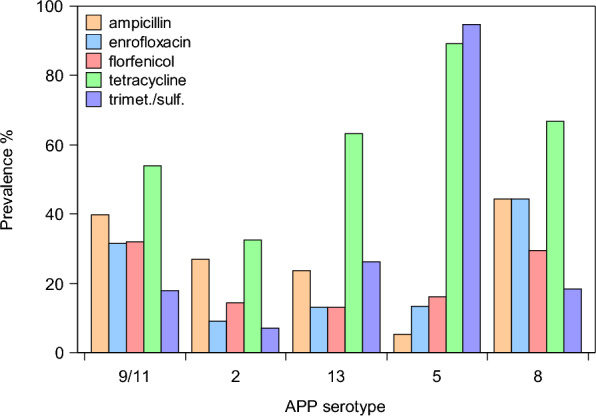
**Prevalence of AMR by serotype.** Prevalence of antimicrobial resistance by serotype in the APP isolates examined during the study period. Only the most common serotypes (i.e., total prevalence > 5%) and resistances (i.e., total prevalence > 10%) are shown.

Resistance of APP isolates to ampicillin (χ^2^_4_ = 16.1; *p* = 0.003), enrofloxacin (χ^2^_4_ = 25.7; *p* < 0.0001), florfenicol (χ^2^_4_ = 15.1; *p* = 0.004), tetracycline and trimethoprim/sulfamethoxazole (χ^2^_4_ = 42.4; *p* < 0.0001) all varied by serotype (Figure 4). Isolates belonging to serotype 9/11 were significantly more resistant to florfenicol than serotypes 2, 13 and 5. Resistance to enrofloxacin was observed more frequently in association with serotype 9/11 and 8, and was less common in 2 and 5. Compared with the other serotypes, serotype 5 rarely showed ampicillin resistance, but conversely was the serotype most commonly associated with tetracycline and trimethoprim/sulfamethoxazole resistance, which were instead rarely encountered in serotype 2 isolates. Resistance to all the other antimicrobials tested was instead independent of serotype and equally likely (*p* > 0.05).

**Figure 5:**
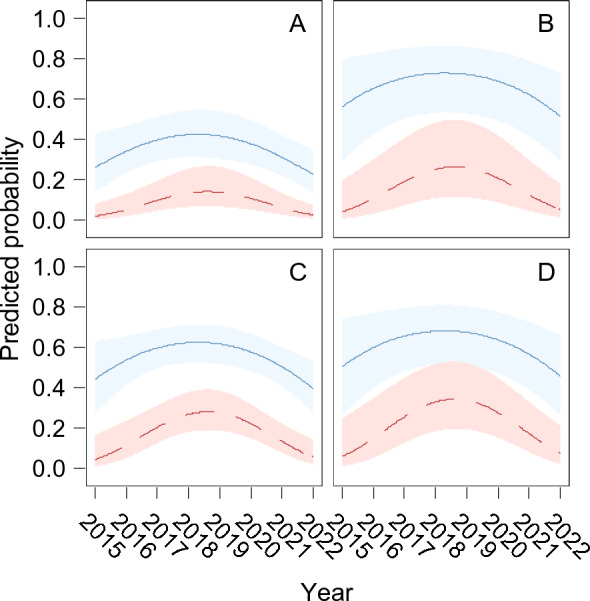
**Resistance to tetracycline and trimethoprim/sulfamethoxazole across years.** Temporal trends in resistance to tetracycline (blue, solid line) and trimethoprim/sulfamethoxazole (red, dashed line) by APP serotypes 9/11 (**A**), 2 (**B**), 13 (**C**) and 8 (**D**). Bands represent the 95% confidence limits of the prediction.

None of the resistances showed any linear variation over time during the study period, although a tendency (*p* = 0.053) for florfenicol resistance to increase can be observed starting from 2020 onwards. However, resistance to tetracycline (χ^2^_1_ = 6.0; *p* = 0.016) and to trimethoprim/sulfamethoxazole (χ^2^_1_ = 10.8; *p* = 0.0013) showed a curvilinear temporal trend: both peaked around 2019 regardless of serotype, and decreased again in the following years (Figure 5).

## Discussion

We retrospectively evaluated the serotypes and AMR profiles of more than 500 APP strains isolated from outbreaks that occurred over 8 years in farms located in the main Italian pig production area (Figure 1). Our analysis revealed that the serotype diversity of APP strains circulating in the area under investigation has increased in recent years, and that AMR is strongly serotype-dependent, highlighting that APP serotyping may help to select the appropriate antimicrobial therapies and improve antimicrobial stewardship.

Our data showed that, although most of the strains causing APP outbreaks in northern Italy belong to serotypes 9/11 and 2, several other variants occasionally occur, especially in the areas where pig density and movements are higher. Overall, serotype 9/11 was the most prevalent (39%), followed by serotype 2 (28%). This result is in contrast with previous studies carried out in the Czech Republic, Germany and Spain, where serotype 2 was the most common variant [9]. Indeed, the distribution of APP serotypes is known to be highly variable in space and time [21, 22]. For instance, in Switzerland, only 7% of strains belonged to serotype 9/11, with the most common serotypes being 7 and 12 [17], which were instead rarely found in our study.

Regarding the less common serotypes in our study, serotype 8 appeared in 2017, while serotype 5, which is a common serotype in other parts of the world (e.g., Korea [23] and Canada [11]), increased in prevalence during the study period: from 3.5% in 2015 to 10% in 2021. Three new serotypes have recently been described, serotypes 17 and 19 in Europe and Canada, whereas serotype 18 has only been found in Europe [6, 24]. While we did not find any APP strains belonging to serotypes 17 or 19, we identified a strain belonging to serotype 18 (Table 1) from an outbreak that occurred in 2022 on a farm in the Lombardy region. Notably, approximately 13% of our isolates was untypable, possibly due to genetic mutations within primers annealing regions of known serotypes, but also potentially indicating new serotypes. Although investigating more in depth these untypable isolates went beyond the scope of the present study, it certainly deserves further attention in the future.

Preserving the efficacy of antimicrobials is crucial for both human and veterinary medicine. In particular, certain classes of antimicrobials have been identified as the highest priority critically important antimicrobials (HPCIAs) for human medicine by the WHO [25]. Some of these (i.e. third- and fourth-generation cephalosporins, quinolones, and polymyxins) have also been included in category B “Restrict” by the European Medicines Agency [26]. These antimicrobials should only be used in the absence of effective alternatives in less critical categories [27]. This is particularly important considering that resistance can be transferred from animals to humans, either directly or through the environment [27]. Regarding the antimicrobial susceptibility of APP, a wide range of antimicrobials are effective against the pathogen, although an increase in resistance to non-critical antimicrobials such as tetracyclines, ampicillin and sulphonamides has been observed in recent years [28]. More than half of our isolates were resistant to tetracycline, a result similar to that reported in other countries such as Canada [15] and Spain [4]. In agreement with previous studies, we also found a relatively low number of isolates resistant to third-generation cephalosporins (i.e., ceftiofur), pleuromutilins (i.e., tiamulin) and macrolides (i.e., tilmicosin, tildipirosin, tulathromycin) [15]. Regarding the aminopenicillins, resistance to amoxicillin/clavulanic acid was rare, but more than 30% of the isolates were resistant to ampicillin. Notably, resistance to ampicillin was widespread among isolates belonging to all serotypes, with prevalence ranging from 20 to 40%. The only exception was serotype 5, which still appeared to be highly susceptible to this antimicrobial. However, isolates belonging to serotype 5 showed concerning levels of resistance (i.e., over 80%) to tetracycline and trimethoprim/sulfamethoxazole.

A previous study investigating AMR in APP isolates was carried out in Italy from 1994 to 2009 [29]. Although comparisons with it should be made with caution, as the authors used the disk diffusion method while we used MICs to estimate AMR, the frequent resistance of APP to tetracycline, ampicillin and trimethoprim/sulfamethoxazole detected in our study is consistent with the patterns described for the previous decade. Conversely, compared to their data, a relative decrease in the proportion of isolates resistant to tiamulin, tulathromycin and tilmicosin, and a parallel increase in resistance to florfenicol and enrofloxacin can be observed [29].

The in vitro activity of florfenicol against clinical isolates of APP has been studied extensively and low resistance levels have been found in Germany, South Korea, Spain and Japan [4, 30–32]. Conversely, we observed a relatively high number of isolates resistant to florfenicol, mainly belonging to serotypes 9/11 and 8, confirming the importance of continuous monitoring of clinical isolates to preserve the efficacy of this antimicrobial. Notably, the higher proportion of isolates resistant to florfenicol and enrofloxacin, an HPCIA and a category B antimicrobial, belonged in both cases to serotype 9/11, which is also the most widespread serotype in the area and one of the most virulent. Even though the resistance rate of APP isolates to most of the antimicrobials tested was relatively stable during the study period, our data revealed curvilinear patterns of susceptibility to tetracycline and trimethoprim/sulfamethoxazole. The proportion of resistant isolates peaked around 2019, regardless of serotype. We also observed a tendency for increased resistance to florfenicol towards the end of the study period which, although not significant, should be kept under close surveillance. Italian sales data of veterinary antimicrobials for the period 2010–2021 show a reduction in sales of tetracyclines and sulphonamides starting around 2016, accompanied by a 26% increase in amphenicols sales, which might explain the observed variations in resistant isolates over the years [33, 34]. However, although pig production can be a major driver of antimicrobial consumption [35, 36] and is the second-largest livestock sector in Italy [33], these data should be interpreted with caution as they cover the whole Italian livestock production, making it challenging to draw conclusions regarding a specific sector. Multiple drug resistance was stable over time but widespread, with more than 30% of the isolates resistant to at least three different antimicrobial classes, two of which were resistant to all nine classes tested. The prevalence of MDR was serotype-dependent. In particular, the highest prevalence was found in serotype 9/11 (46%), which was almost three times higher than the one with the lowest prevalence, serotype 2 (17%).

To the best of our knowledge, this is the first study attempting to detail the APP serotypes circulating in Italy and to associate them with antimicrobial resistance. However, the study focuses on northern Italy, which is the area where most of Italian pig production is concentrated, and this does not allow us to exclude that different patterns may occur in central and southern Italy. In addition, our analysis was limited by the lack of information about treatment history of the sampled animals, as well as a lack of data regarding herd vaccination, both of which could have altered detection rate. Despite these limitations, the different AMR profiles of serotypes and their temporal changes highlight the need to rely on detailed diagnostic data to control the disease more effectively and preserve the efficacy of antimicrobials, preventing the emergence of clinical resistances. Moreover, as previously mentioned, the genetic and phenotypic diversity of the different serotypes of APP hinders the development of a broadly protective vaccine covering all serotypes [37, 38]. Distinguishing between the 19 serotypes of APP might be therefore relevant not only for disease management, but also for the production of geographically relevant vaccines [14, 22].

In conclusion, the evaluation of over 500 APP strains from several Italian pig farms revealed an increasing serotype diversity, with serotype 9/11 being the most prevalent, and serotype-dependent AMR patterns. Our study also identified emerging serotypes, such as 8, and an increase in serotype 5 prevalence. Resistance to tetracycline was widespread while resistances to critical antimicrobials, such as third-generation cephalosporins, were still relatively low. Continuous monitoring is crucial, as resistant isolates showed curvilinear patterns, and serotype 9/11 exhibited the highest MDR prevalence. These findings underscore the need for detailed diagnostic data and antimicrobial stewardship to curb the infection and also preserve antimicrobial efficacy on the long term. Particularly, considering the potential transfer of resistances from animals to humans.

### Supplementary Information

**Additional file 1.** **Epidemiological cut-off values.** This additional file includes a table listing the EUCAST epidemiological cut-offs (ECOFFs) used to evaluate antimicrobial resistance in *A. pleuropneumoniae* isolates.

**Additional file 2.** **Distribution of outbreaks by farm.** This additional file includes a figure illustrating the no. of outbreaks per farm analysed during the study period.

## Data Availability

The datasets generated and/or analysed during the current study are available from the corresponding author on reasonable request.
