# Supplementary material for: Serotype diversity and antimicrobial susceptibility profiles of Actinobacillus pleuropneumoniae isolated in Italian pig farms from 2015 to 2022
Source: Vet Res. 2024 Apr 9;55:48. doi: 10.1186/s13567-024-01305-x (PMC11005290; doi:10.1186/s13567-024-01305-x)
Supplement: Supplementary file 1 — Additional file 1. Epidemiological cut-off values. This additional file includes a table listing the EUCAST epidemiological cut-offs (ECOFFs) used to evaluate antimicrobial resistance in A. pleuropneumoniae isolates. [file 13567_2024_1305_MOESM1_ESM.docx]

**Additional file 1** **EUCAST epidemiological cut-offs for *Actinobacillus pleuropneaumoniae* (ECOFFs, accessed in March 2024) relative to the tested antimicrobials.**

| **Antimicrobial** | **ECOFF (R > mg/L)** |
| --- | --- |
| Amoxicillin/clavulanic | 1 |
| Ampicillin | 0.5 |
| Ceftiofur | 0.06 |
| Enrofloxacin | 0.125 |
| Florfenicol | 1 |
| Tetracycline | 2 |
| Tiamulin | 32 |
| Tildipirosin | 16 |
| Tilmicosin | 32 |
| Trimethoprim/Sulfamethoxazole | 0.25 |
| Tulathtromicin | 64 |
