# Supplementary material for: Serotype diversity and antimicrobial susceptibility profiles of Actinobacillus pleuropneumoniae isolated in Italian pig farms from 2015 to 2022
Source: Vet Res. 2024 Apr 9;55:48. doi: 10.1186/s13567-024-01305-x (PMC11005290; doi:10.1186/s13567-024-01305-x)
Supplement: Supplementary file 2 — Additional file 2. Distribution of outbreaks by farm. This additional file includes a figure illustrating the no. of outbreaks per farm analysed during the study period. [file 13567_2024_1305_MOESM2_ESM.docx]

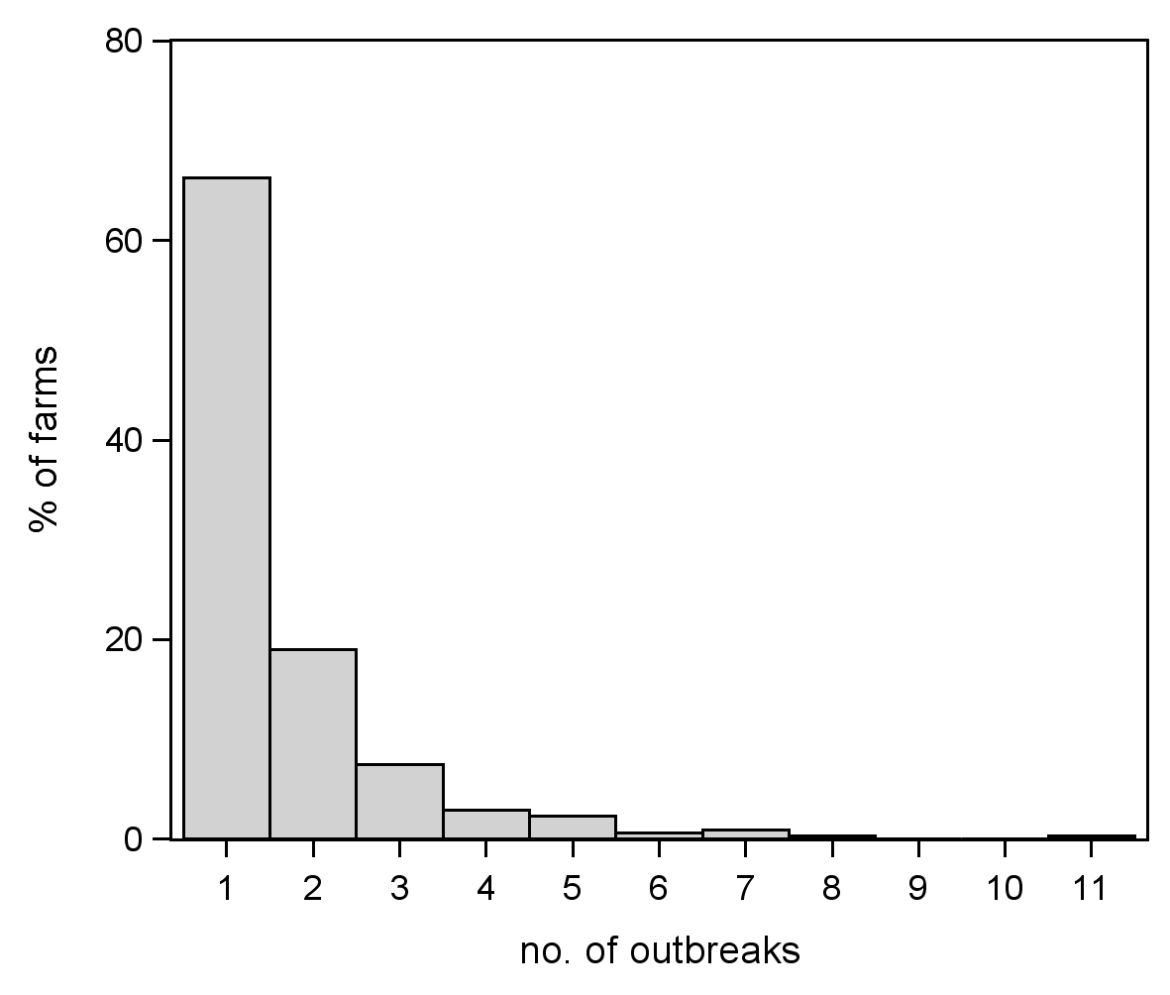


**Additional file 2 Histogram illustrating the frequency distribution of *Actinobacillus pleuropneumoniae* outbreaks in North Italian pig farms from 2015 to 2022.**
